# Octahedral Ni-nanocluster (Ni_85_) for Efficient and Selective Reduction of Nitric Oxide (NO) to Nitrogen (N_2_)

**DOI:** 10.1038/srep25590

**Published:** 2016-05-09

**Authors:** Arup Mahata, Kuber Singh Rawat, Indrani Choudhuri, Biswarup Pathak

**Affiliations:** 1 Discipline of Chemistry, School of Basic Sciences, Indian Institute of Technology (IIT) Indore, Indore, M.P., India; 2 Center for Material Science and Engineering, Indian Institute of Technology (IIT) Indore, Indore, M.P., India

## Abstract

Nitric oxide (NO) reduction pathways are systematically studied on a (111) facet of the octahedral nickel (Ni_85_) nanocluster in the presence/absence of hydrogen. Thermodynamic (reaction free energies) and kinetic (free energy barriers, and temperature dependent reaction rates) parameters are investigated to find out the most favoured reduction pathway for NO reduction. The catalytic activity of the Ni-nanocluster is investigated in greater detail toward the product selectivity (N_2_ vs. N_2_O vs. NH_3_). The previous theoretical (catalyzed by Pt, Pd, Rh and Ir) and experimental reports (catalyzed by Pt, Ag, Pd) show that direct N-O bond dissociation is very much unlikely due to the high-energy barrier but our study shows that the reaction is thermodynamically and kinetically favourable when catalysed by the octahedral Ni-nanocluster. The catalytic activity of the Ni-nanocluster toward NO reduction reaction is very much efficient and selective toward N_2_ formation even in the presence of hydrogen. However, N_2_O (one of the major by-products) formation is very much unlikely due to the high activation barrier. Our microkinetic analysis shows that even at high hydrogen partial pressures, the catalyst is very much selective toward N_2_ formation over NH_3_.

Reduction of NO has attracted considerable attention in recent years due to the environmental pollutions and industrial applications^1^,^2^. NO is produced as a by-product during the combustion of hydrocarbons and is responsible for environmental (such as photochemical smog, acid rain, ozone depletion) and biological problems^3^,^4^,^5^. Therefore, the removal of NO is highly sought after and a major challenge for researchers. Extensive progress has been made over Pt^6^,^7^,^8^,^9^,^10^,^11^,^12^, Pd^13^,^14^,^15^, Rh^16^,^17^,^18^,^19^,^20^ and Ir^21^,^22^,^23^ metal surfaces for the catalytic conversion of NO to N_2_. However, the N-O bond dissociation is one of the most important steps and N-O can be dissociated via two pathways (i) direct N-O bond dissociation or (ii) indirect (hydrogenation followed by N-O bond dissociation). Earlier studies^6^,^7^,^8^,^9^,^10^,^11^,^12^,^13^,^14^,^15^,^16^,^17^,^18^,^19^,^20^,^21^,^22^,^23^ reported that direct N-O bond dissociation is not thermodynamically favourable over Pt^6^,^7^,^8^,^9^,^10^,^11^,^12^, Pd^13^,^14^,^15^, Rh^16^,^17^,^18^,^19^,^20^ and Ir^21^,^22^,^23^ metal surfaces, but possible from their hydrogenated products (NOH, HNO, HNOH, H_2_NO), which increases the possibility of unwanted by-products. Therefore, the relative selectivity of N_2_ vs. other products (N_2_O and NH_3_) varies from catalyst to catalyst. The reduction selectivity toward N_2_ formation is more dependent on the direct N-O bond dissociation kinetics. Hence the main objective of this work is to design an efficient catalyst to improve the N-O bond dissociation kinetics to increase the N_2_ production selectivity.

NO reduction reactions have been studied mainly over precious metal based catalysts. However, considerable efforts have been made to reduce the cost of metal catalysts and among all the low-cost based metal-catalysts, Ni is the most effective catalyst due to the easy availability of valence shell d-electrons^24^,^25^,^26^,^27^,^28^,^29^,^30^. Experimental and theoretical studies have been carried out for NO reduction reactions catalysed by Ni-catalysts^31^,^32^,^33^,^34^,^35^,^36^,^37^, and it is reported that NO undergoes dissociative adsorption over Ni-catalyst^38^, resulting in the formation of N_2_ as a major product. However, the role of hydrogen (reducing agent) is not considered in any of these studies, and thus a vis-à-vis comparison cannot be made between the direct and indirect N-O bond activation processes. Therefore, it is very important to find out whether Ni-catalyst could trigger the direct N-O bond dissociation or favours the indirect N-O bond dissociation as reported for noble metal based catalysts^6^,^7^,^8^,^9^,^10^,^11^,^12^,^13^,^14^,^15^,^16^,^17^,^18^,^19^,^20^,^21^,^22^,^23^.

Apart from different metal based catalysts, the surface morphology and size of the metal catalyst play important roles in the bond scission process, which in turn control the catalytic activity of a metal catalyst. In this context, metal nanoclusters surrounded by well-defined facets show better catalytic activities compared to their bulk surfaces. Metal nanoclusters enclosed by multi-facets are very noble catalysts and are reported to be very promising for electro-catalytic reactions^39^,^40^,^41^ due to the presence of high surface unsaturation. Many theoretical studies have been reported on small size metal nano-clusters^42^,^43^,^44^,^45^ to understand their catalytic activities toward nitric oxide reduction. However, the size of the nanoclusters is very important for catalytic reactivity due to their finite-size effects^46^,^47^,^48^,^49^,^50^,^51^ and well-defined facets. These low-coordinated sites have higher d-band energies, which actually increase their reactivity^42^,^43^. To the best of our knowledge, there are no reports on NO reduction on a well-defined nanocluster based catalyst. Therefore, to understand the activity of the nanocluster, it is necessary to model a nanocluster surrounded by well-defined facets. The nanocluster with octahedral shape is one of the most stable forms due to its high symmetry^52^,^53^. In fact, the Ni(111) surface is mainly observed in the experimentally synthesized Ni-nanoclusters^54^,^55^,^56^. Hence we have modelled a ~1 nm size of octahedral nickel nanocluster (Ni_85_) enclosed by well-defined low index facets [eight (111) facets] to understand the NO reduction activity (Fig. 1).

It is also very important to study the complete catalytic process in the presence and absence of hydrogen to understand the underlying NO reduction reaction mechanism to improve the product selectivity. The N_2_ production selectivity can be significantly improved if the reduction mechanism goes through the following two steps: direct NO dissociation [*NO → *N + *O] followed by N_2_ formation [*N + *N → *N_2_]. Species are denoted with an asterisk (*) while adsorbed on the surface. But then there are possibilities of by-product formation, such as N_2_O formation: *NO + *N → *N_2_O. Koper *et al*.^57^ reported that NH_3_ is formed via an indirect N-O bond dissociation process. In that case hydrogenation of NO (formation of *HNO and *H_2_NO intermediates) followed by N-O bond dissociation favours the formation of NH_3_. Cuesta *et al*.^58^ found *HNOH as the predominant intermediate for NH_3_ formation. Previous theoretical studies reported Ir(211) selectively produces N_2_, while Pt(211) produces^8^ N_2_ and N_2_O but they did not consider the role of hydrogen. Carrie *et al*.^6^ studied NO reduction on the Pt (111) surface to understand the N_2_O formation as a major product observed experimentally at low temperatures^59^,^60^. In spite of several studies, direct N-O bond dissociation is calculated to be the least favourable pathway in the presence/absence of hydrogen gas.

Here we have systematically studied the complete NO reduction pathways on a Ni(111) facet of the nanocluster to find out the most favoured pathway(s) to control the product selectivity. Microkinetic analysis is performed to understand the role of temperature and partial pressure towards the product selectivity. The catalytic activity of the nanocluster is compared with the bulk Ni(111) surface to have a vis-à-vis comparison for the NO reduction reaction. For comparison with a noble metal catalyst, the catalytic activity is compared with that of bulk Pt(111). An attempt has been made to understand the excellent catalytic activity of the nanocluster toward NO reduction and product selectivity compared to any other catalysts reported previously.

## Results

### Adsorption

**Figure 1:**
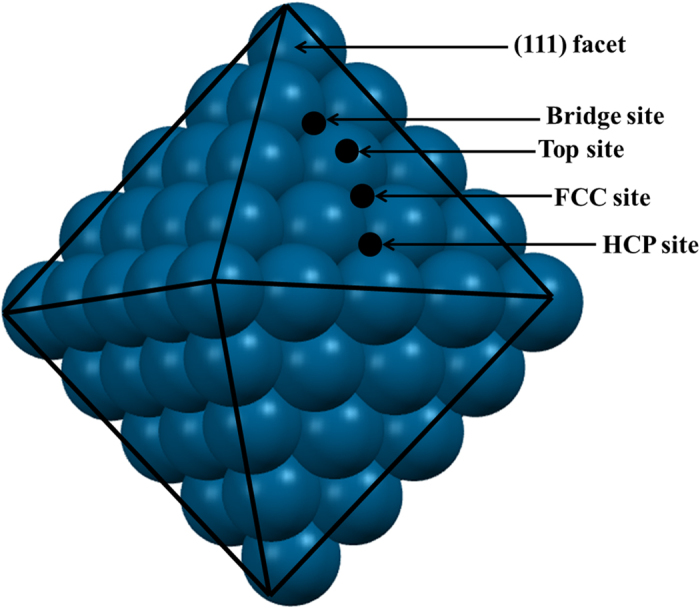
Octahedral Ni_85_ nanocluster enclosed by eight (111) facets.

Four different catalytic sites (Fig. 1) are present on the (111) facet of the nanocluster: (i) top, (ii) bridge, (iii) hexagonal close packed (hcp) and (iv) face centred cubic (fcc). The relative stabilities of the adsorbed conformers are studied at four possible sites and the most stable conformer is considered for further study.

**Figure 2:**
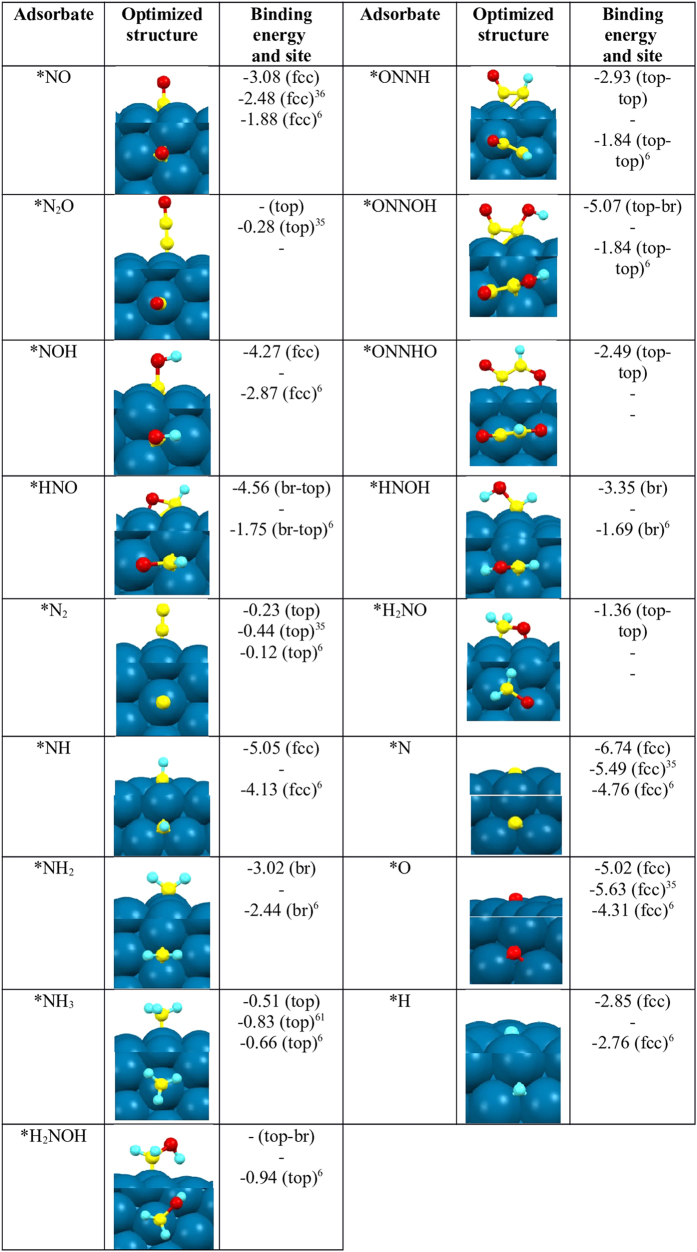
Site preference, binding energy (eV) of the most stable adsorbates on the (111) facet of the Ni_85_ naocluster. The sites preferences and binding energies of the respective adsorbates are compared with previous reports on Pt(111)^6^ and Ni(111)^35^,^36^,^61^ surfaces. Yellow, cyan, red, and blue spheres represent N, H, O, and Ni, respectively.

Intermediates adsorbed on the Ni(111) facet of the nanocluster are presented in Fig. 2. The most preferred binding sites of the intermediates and their respective binding energies are presented (Fig. 2) and compared with previous theoretical studies on Ni(111)^35^,^36^,^61^ and Pt(111)^6^ surfaces. The adsorption energies (E_ad_) for all possible adsorbates are calculated using the following equation:


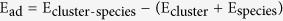


where E_cluster-species_ is the total energy of the cluster with adsorbed species, E_cluster_ is the energy of the nickel cluster and E_species_ is the energy of the intermediate species. The binding energies are in very much agreement with previous studies^6^,^7^,^35^,^36^,^37^ though some of our calculated binding energies are higher than the previous reported values. The differences in adsorption energies compared to previous theoretical values could be due to the excellent catalytic activity of the Ni-nanocluster. The adsorption patterns of some of the important intermediates (*NO, *N_2_O, *N_2_, *NOH, *HNO, *H_2_NOH and *NH_3_) are discussed and compared to previous experimental and theoretical reports.

### *NO

*NO prefers to be adsorbed at the fcc site (Fig. 2) of Ni(111) through its N atom with an adsorption energy of −3.08 eV. Wu *et al*.^35^ reported that the *NO molecule prefers to occupy the fcc site of the Ni(111) surface with an adsorption energy of −2.46 eV. It has been experimentally^38^ as well as theoretically^35^,^62^ reported that NO prefers to be adsorbed through its N atom as it has more vacant orbitals than an O-atom. The vibrational frequency of the *N-O bond (1460–1543 cm^−1^) while adsorbed at the fcc site of Ni(111) surface is characterized^38^ using RAIRS, EELS and PED, and agrees well with our calculated value of 1530 cm^−1^. Therefore, our results are in very much agreement with previous reports on binding energy and vibrational frequency^35^,^36^,^37^.

### *N_2_O

*N_2_O prefers (Fig. 2) to be adsorbed at a top site of the Ni(111) facet. A theoretical study^63^ on the (111) terrace of the high index Ni(755) surface reported a <N-N-O bond angle of 180° and Ni-N, N-N and N-O bond distances of 1.84, 1.15 and 1.20 Å, respectively. They are in very much agreement with our calculated (Fig. 2) <N-N-O bond angle of 179.66° and Ni-N, N-N and N-O bond distances of 1.83, 1.14, 1.20 Å respectively. Using XPS and TPD studies, they reported that *N_2_O weakly adsorbed on the Ni(111) surface through its N-atom^64^,^65^. Thus, the calculated structural parameters are in very much agreement with the previous report^35^. This suggests that our study on the Ni(111) facet is in very close agreement with previous experimental reports on the high index Ni(755) surface^64^,^65^.

### *NOH and *HNO

In the presence of hydrogen, *NO can react with *H to form *NOH and *HNO. *NOH adsorbs strongly at the fcc-site (Fig. 2) through its N atom with adsorption energy of −4.27 eV. On the other hand, *HNO binds in a di-sigma manner (Fig. 2) with an adsorption energy of −4.56 eV. Their binding preferences are very much in agreement with previous findings^6^,^7^,^13^.

### *N_2_

*N_2_ binds perpendicular to the top site of the (111) facet of the nanocluster (Fig. 2) with adsorption energy of −0.23 eV. The calculated Ni-N and N-N bond distances are 1.78 Å and 1.12 Å respectively. The N-N bond distance is slightly lengthened while adsorbed on the surface compared to the N-N distance (1.10 Å) in the N_2_ gas molecule, suggesting that the interaction is very weak between the surface and gas molecule. Previous studies^35^,^36^ on the Ni(111) surface reported that the top site is the most favoured position for N_2_ adsorption. Using infrared reflection absorption spectroscopy, Yoshinobu *et al*.^66^ reported that *N_2_ adsorbs perpendicular on the Ni(111) surface with an adsorption energy of −0.36 eV.

### *NHx (x = 1–3)

*NH_x_ containing species (Fig. 2) can be formed during the course of reaction. These intermediates (*NH, *NH_2_ and *NH_3_) are most stable at the fcc, bridge and top sites with Ni-N bond distances of 1.81, 1.91 and 2.01 Å respectively (Fig. 2). *NH and *NH_2_ bind strongly, whereas *NH_3_ binds weakly on the Ni-nanocluster surface. The calculated adsorption energies are −5.05 eV, −3.02 eV and −0.51 eV for *NH, *NH_2_ and *NH_3_ respectively. Sergey *et al*.^61^ reported *NH_3_ prefers to bind on the top site of the Ni(111) surface, which agrees well with our finding. The weak adsorption of *NH_3_ suggests the easy desorption of NH_3_ from the catalyst surface, which in turn reduces the possibility of surface poisoning.

### *H_2_NOH

*H_2_NOH can be formed via hydrogenation at the N- and O- sites of *HNOH and *H_2_NO. *H_2_NOH adsorbs on the top-bridge position by its N- (Ni-N = 1.97 Å) and O atoms (Fig. 2).

It is interesting to find out that adsorption energy decreases (Fig. 2) from NO to higher hydrogenated products implying that the interactions are weaker for higher hydrogenated products.

### Reaction Free Energy and Activation Barriers

The reaction free energies are calculated from the total energy difference between the products and reactants. Thus negative free energy suggests the exergonic nature of the reaction, whereas positive reaction energy suggests the endergonic nature of the reaction. Activation barriers are calculated from the energy differences between the transition and initial states.

### *N_2_ and *N_2_O Formation

**Figure 3:**
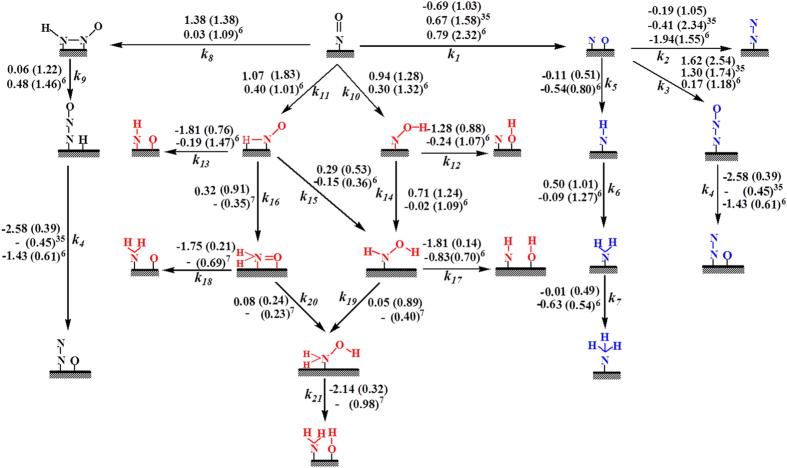
Reaction Scheme. Reaction free energies (eV) and activation barriers (eV, in parenthesis) are presented for all the possible elementary steps for NO reduction over the (111) facet of the Ni_85_ nanocluster. Our calculated respective values are compared with previous reports on NO reduction over Ni(111)^35^ and Pt(111)^6^,^7^ bulk surfaces.

The direct N-O bond dissociation (Fig. 3) can lead to the formation of important products such as *N_2_, and *N_2_O.


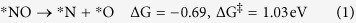



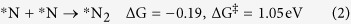



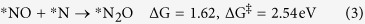



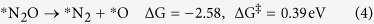


The direct N-O bond dissociation (step 1) is a downhill process (−0.69 eV) with an activation barrier of 1.03 eV. The *N-O bond distance is 1.22 Å, which is higher than the N-O bond distance (1.15 Å) in the gas (NO) molecule. This suggests that the gas molecule gets activated during the adsorption process. The previously calculated N-O bond dissociation barriers are 1.49 eV^36^ and 1.58 eV^35^ on Ni(111) surfaces, which are far higher than our calculated barrier of 1.03 eV. In fact, the higher *N-O bond distance (1.22 Å), compared to previous report (1.18 Å)^37^, suggests the excellent catalytic activity of the nanocluster. Earlier reports of N-O bond dissociation barriers on bulk Pt(111) surfaces are 2.60 eV^8^ and 2.32 eV^6^, respectively, which are considerably higher than our calculated barrier of 1.03 eV. Therefore, Ni_85_ nanocluster is certainly an efficient catalyst for the direct N-O bond activation reaction.

The formation of *N_2_ (step 2) from the atomic nitrogens (*N) is exergonic by −0.19 eV with an activation barrier of 1.05 eV. Wu *et al*.^35^ reported an activation barrier of 2.34 eV for the same step on the bulk Ni(111) surface. Carrie *et al*.^6^ studied the *N_2_ formation on Pt(111) surface and reported a free energy barrier of 1.55 eV. Therefore, the Ni_85_ nanocluster shows excellent catalytic activity toward *N_2_ formation compared to noble metal based catalysts.

*N_2_ formation is possible via another pathway where *N_2_O (step 3) formation takes place, followed by *N_2_O dissociation (step 4). The *N_2_O formation is highly endergonic (1.62 eV) with a free energy barrier of 2.54 eV, whereas *N_2_O dissociation is highly exergonic (−2.58 eV) with an activation barrier of 0.39 eV. Wu *et al*.^35^ reported the *N_2_O formation barrier of 1.74 eV (step 3) on the Ni(111) surface. Carrie *et al*. reported the *N_2_O formation and dissociation barriers^6^ of 1.18 eV and 0.61 eV on Pt(111). The calculated barrier for *N_2_O formation is significantly higher than the previous reports on Ni(111) and Pt(111) surfaces.

Wu *et al*.^35^ studied the first three steps of NO reduction on the Ni(111) surface as follows: (i) *NO dissociation, (ii) *N_2_ and (iii) *N_2_O formation. The calculated activation barriers are 1.58 eV (*NO dissociation), 2.34 (*N_2_ formation) and 1.74 eV (*N_2_O formation) respectively. Similarly Carrie *et al*.^6^, reported activation barriers of 2.32 eV (*NO dissociation), 1.55 eV (*N_2_ formation), 1.18 eV (*N_2_O formation), and 0.61 eV (*N_2_O dissociation) over Pt(111). Therefore, the previous reports show *N_2_O formation is favourable over *N_2_ formation, whereas our study shows *N_2_ formation is favourable over *N_2_O formation. However, *N_2_O dissociation is very favourable (step 4) in all the cases. But on the Ni_85_ cluster’s surface, the *N_2_O formation is very unlikely to happen due to the high activation barrier of 2.54 eV. Therefore, our nanocluster shows excellent catalytic activity toward *N_2_ formation, compared to earlier reports^6^,^8^,^35^,^36^,^37^. Hence, the dissociation of the N-O bond followed by N-N bond formation (*NO → *N → *N_2_) is the most favourable pathway in the absence of hydrogen.

### *NH_3_ Formation


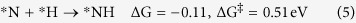



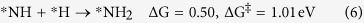



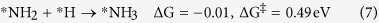


Hydrogen is used as a reducing agent for NO reduction. The dissociation of hydrogen toward atomic hydrogens (*H_2_ → *H + *H) is highly exergonic (−1.07 eV) with an activation barrier of 0.02 eV. The dissociative adsorption of hydrogen on the Pt (111) bulk surface is reported to be exergonic by −0.87 eV with an activation barrier of 0.00 eV^6^. The activation barrier for hydrogen dissociation on Ni(111) surface is very much comparable with that on the Pt(111) surface. However, the reaction is highly exergonic on the Ni-surface compared to the Pt(111) surface^25^,^67^. Therefore, the Ni-nanocluster is a promising candidate for the hydrogenation reaction.

The first hydrogenation on *N (step 5, *NH formation) has an activation barrier of 0.51 eV though the reaction is exergonic by −0.11 eV. In contrast, the second hydrogenation (step 6) for the formation of *NH_2_ is endergonic by 0.50 eV with an activation barrier of 1.01 eV. The formation between *NH_2_ (step 6) and *N_2_ (step 2) can be very competitive as both the reaction steps (Fig. 3) have comparable free energy barriers (1.01–1.05 eV). However, the third hydrogenation (step 7, *NH_3_ formation) has an activation barrier of 0.49 eV with reaction free energy of −0.01 eV, suggesting that the *NH_3_ formation is very much favourable.

Hence, in the presence of hydrogen, *NO dissociation could lead to competition between *N_2_ and *NH formation. The calculated activation barriers show that *NH formation (barrier = 0.51 eV) is more favourable than *N_2_ formation (barrier = 1.05 eV). However, *NH_2_ formation (barrier = 1.01 eV) is not much favourable compared to *N_2_ formation (barrier = 1.05 eV). But, our reaction thermodynamics shows, *N_2_ formation is highly exergonic (−0.19 eV) compared to *NH_2_ formation (0.50 eV). Therefore, *N_2_ formation is a spontaneous process where as *NH_2_ formation is not thermodynamically favourable. Even the *NH (*NH → *N + *H) dissociative barrier (Fig. 3) is calculated to be 0.62 eV with a reaction energy of 0.11 eV, indicating the tendency of *NH to decompose to *N instead of hydrogenation to *NH_2_. Therefore *N_2_ formation is very much favourable, compared to ammonia formation even in the presence of hydrogen.

The hydrogenation at *NO on the Pt(111) surface leads to the indirect dissociation of the N-O bond, which in turn reduces the selectivity of *N_2_ formation over *NH_3_ formation^68^,^69^. Surprisingly, we find that the direct N-O bond dissociation barrier is lower compared to the indirect (*N-OH and *HN-O) bond dissociation barrier on the Ni(111) facet of the nanocluster. Therefore, on the Ni-nanocluster surface, *NO can be reduced to *N_2_ even in the absence of hydrogen, which minimizes the possibility of unwanted by-products (*NH_3_ and *N_2_O) formation.

*N_2_O can also be formed (Fig. 3) by the direct combination of *NO and *NH (step 8) followed by the dissociation of *ONNH (step 9).


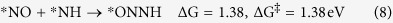



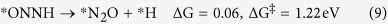


The formation of *ONNH from *NO and *NH (step 8), is highly endergonic (1.38 eV) with an activation barrier of 1.38 eV. However, the dissociation of *ONNH (step 9) leads to the formation of *N_2_O, which is mildly endergonic (0.06 eV), with an activation barrier of 1.22 eV. But *N_2_O dissociation is a spontaneous process with an activation barrier of 0.39 eV (step 4). Thus, *N_2_O formation is very unlikely due to the high activation barrier.

### *NO Hydrogenation

#### First Hydrogenation and Dissociation steps


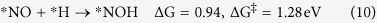



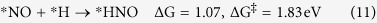



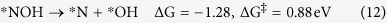



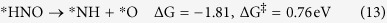


In the hydrogenation pathways (Fig. 3), *NO can be hydrogenated to form *NOH and *HNO respectively. Here, we have studied all the hydrogenated intermediates (Fig. 3) to find out the thermodynamic and kinetic feasibility of such reactions.

The *NO hydrogenation reactions (step 10 and 11) are endergonic by 0.94 eV (step 10) and 1.07 eV (step 11) with activation barriers of 1.28 eV (step 10) and 1.83 eV (step 11), respectively. Therefore, hydrogenation at the O atom is more favourable over the N atom. The relative stabilities of the isomers (*NOH and *HNO) show that *NOH is more stable (by 0.15 eV) over *HNO. Previous reports also show that NO undergoes hydrogenation at O-^7^ and N- atoms^6^ though favourable at the O atom.

The reaction energies for N-O bond dissociation of the hydrogenated species are −1.28 eV (step 12) and −1.81 eV (step 13) and activation barriers are 0.88 eV and 0.76 eV for *NOH and *HNO respectively. Therefore, hydrogenation on NO (Fig. 3) lowers the N-O bond dissociation barrier. Our study shows that *HNO has a lower N-O bond dissociation barrier than *NOH. As *NH and *N are the two competitive intermediates for *NH_3_ and *N_2_ formation, therefore N-O dissociation barriers of *HNO and *NOH give an idea about the selectivity of product formation. Carrie *et al*.^6^ reported that the dissociation barrier of the N-O bond is less in *NOH than in *NO on the Pt (111) surface, suggesting that *N_2_O is the predominant product. In fact, all the previous studies^6^,^7^ show indirect N-O bond dissociation is favourable over direct N-O bond dissociation, whereas we find direct N-O bond dissociation is favourable over indirect N-O bond dissociation. Therefore, the Ni_85_ nanocluster is an efficient and a selective catalyst for *N_2_ formation over *N_2_O and *NH_3_ formation.

#### Second Hydrogenation and Dissociation steps


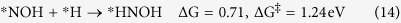



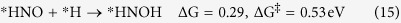



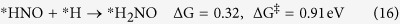


The *NOH can further undergo hydrogenation for the formation of *HNOH (step 14). It has an activation barrier of 1.24 eV with reaction free energy of 0.71 eV. Similarly *HNO can undergo hydrogenation for the formation of *HNOH (step 15) and *H_2_NO (step 16). *HNOH formation is endergonic by 0.29 eV with an activation barrier of 0.53 eV and *H_2_NO formation is endergonic by 0.32 eV with an activation barrier of 0.91 eV. Hence, hydrogenation on *HNO is thermodynamically and kinetically favourable over hydrogenation on *NOH.


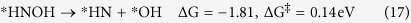



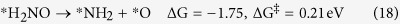


Now, both the hydrogenated intermediates (*HNOH and *H_2_NO) can undergo N-O bond dissociation to produce *NH and *NH_2_ respectively (step 17–18). The dissociation reactions (step 17–18) are highly exergonic and have low activation barriers and are easy to dissociate.

Our results show that *NO prefers to be hydrogenated at the O-centre to form *NOH followed by hydrogenation at the N-centre to form *HNOH, which support the preference of hydrogenation at the O-centre over the N-centre. The preference of hydrogenation at the O-centre is also found for the first hydrogenation of *NO (*NO + *H → *NOH). Andre *et al*.^7^ also reported that *NOH and *HNOH are the favourable intermediates over *HNO and *H_2_NO intermediates. Therefore, the most favourable pathway for *NH_3_ formation is *NO → *NOH → *HNOH → *NH, whereas the least favourable pathway is *NO → *HNO → *HNOH → *NH.

#### Third Hydrogenation and Dissociation steps


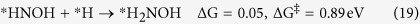



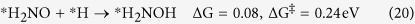


The third hydrogenation (step 19–20) on *NO can take place for the formation of *H_2_NOH and *H_2_NOH. Hydrogenation at the N-centre (step 19) is mildly endergonic by 0.05 eV with an activation barrier of 0.89 eV, whereas hydrogenation at the O-centre (step 20) is mildly endergonic by 0.08 eV with an activation barrier of 0.24 eV. Interestingly, hydrogenation becomes more and more favourable as the number of hydrogens increases.


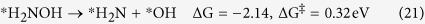


The N-O dissociation from *H_2_NOH is highly exergonic (−2.14 eV, step 21) with an activation barrier of 0.32 eV. However, the N-O bond dissociation barrier is lower in *HN-OH (0.14 eV) than in *H_2_N-O, indicating that *HNOH is likely to dissociate (step 17). Therefore, the N-O bond dissociation barriers are lower and lower for the higher hydrogenated intermediates.

The N-O bond distances in the intermediate species support such a trend. The calculated N-O bond distances are 1.22 Å, 1.40 Å, 1.43 Å and 1.44 Å in *NO, *NOH, *HNOH and *H_2_NOH respectively, suggesting that N-O bond dissociation is very likely for the higher hydrogenated products.

After going through all the possible hydrogenation and non-hydrogenation pathways, we find N_2_ formation is highly favourable over NH_3_ formation. Therefore, the most favourable route toward N_2_ formation is *NO → *N → *N_2_, whereas *NO → *ONNH → *N_2_O → *N_2_ is the less favourable route. The most favourable route toward NH_3_ formation is *NO → *NOH → *HNOH → *NH → *NH_2_ → *NH_3_ and the less favourable routes are *NO → *HNO → *HNOH → *NH → *NH_2_ → *NH_3_ and *NO → *HNO → *HNOH → *H_2_NOH → *NH_2_ → *NH_3_.

#### Non-favourable Pathways

Along with the most favourable pathways, we have studied some other possible pathways, which are discussed as follows.


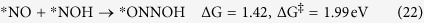



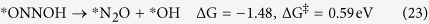



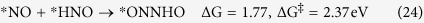



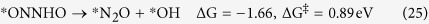


*NOH could react with *NO to form *ONNOH (step 22) with an activation barrier of 1.99 eV. As the barrier is very high, this reaction is very unlikely to proceed. *ONNOH has a dissociation barrier of 0.59 eV for the formation of *N_2_O (step 23). The other possibility is *NO reacting with *HNO for the formation of a N-N bond (step 24; via *ONNHO) followed by N-O bond dissociation (step 25). The N-N bond formation step is highly endergonic by 1.77 eV with an activation barrier of 2.37 eV, and thus is very unlikely to happen. However, the N-O bond dissociation (step 25) is highly exergonic (−1.66 eV) with an activation barrier of 0.89 eV. The calculated activation barriers are 2.37 eV and 0.89 eV for N-N bond formation and N-O bond dissociation steps respectively. The lower activation barrier for the formation of *ONNOH over *ONNHO is due to the stability (by 0.15 eV) and strong adsorption energy (by 2.58 eV) of *ONNOH species compared with *ONNHO. The lower activation barrier for N-O bond dissociation in *ONNOH over *ONNHO is attributed to the longer N-O bond distance (1.42 Å) in *ONNOH than in *ONNHO (1.32 Å). Such higher activation barrier for *ONNHO (2.04 eV) formation over *ONNOH formation (1.42 eV) has been reported previously on the Pt(111) surface^7^.

### Kinetic Analysis

From the elementary pathways, we discover that many pathways are possible for a N-O bond dissociation reaction. The Gibbs free energy versus reaction coordinate gives an overall idea to locate the minimum energy pathway from several possibilities. The roles of surface coverage, gas phase partial pressures of reactant and product and reaction temperature cannot be fully understood from the Gibbs free energy calculations. These experimental parameters can provide further insights to find out the most favoured pathway for the NO reduction reaction. Thus, we have done a detailed microkinetic analysis based on our preliminary DFT results to understand the roles of surface coverage, partial pressures of reactants/products and reaction temperatures toward the reaction kinetics. The forward (k_i_) and backward (k_−i_) rate constants for all the elementary steps are calculated using the following equation:


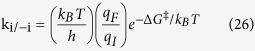


where k_B_ is the Boltzmann constant, T is the temperature, h is the Plank constant. Here q_I_ and q_F_ are vibrational partition functions for the initial and final state structures and ΔG^‡^ is the Gibbs free energy barrier for the initial and final state of the elementary reaction. The vibrational partition functions (q) are calculated using the following equation:


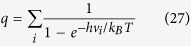


where υ_i_ are the vibrational frequencies. All the exergonic reactions are assumed to be irreversible. Hence only forward steps are considered. For endergonic reactions, both the forward and backward steps are considered while developing the microkinetic model. *k*_*i*_ and *k*_*−i*_ are used for the forward and backward reaction steps respectively for the *i*-th step. The details of the microkinetic model are given in the Supporting Information.

Here, using the mickokinetic model, we would like to understand the role of hydrogen partial pressure toward the hydrogenation of NO. In the presence of high hydrogen partial pressures, we would like to study whether or not *NO will favour hydrogenation over *N-O direct dissociation.

As the experimental NO reduction temperature ranges from 300 K to 500 K^68^,^69^, the rate constants (Table 1) are calculated in the same temperature range (300 K to 500 K). The rate constants improve significantly as we increase the temperature. At 300 K, the ratio between the rate constants (*k*_10_/*k*_11_) is ~10^9^ for *NO hydrogenation at O- and N-sites respectively. Hence, *NOH formation is highly favourable over *HNO formation (*NO + *H → *NOH and *NO + *H → *HNO). The higher possibility of *NOH formation can be further confirmed from the surface coverage (Θ) study. The surface coverage ratio of the Θ_NOH_/Θ_HNO_ (see Supporting Information for details) is calculated using the steady state approximation^70^, where the rates of production and decomposition are assumed to be equal. Therefore, Θ_NOH_ and Θ_HNO_ are calculated considering their equal rate of formation and decomposition in the complete reaction pathway. The Θ_NOH_/Θ_HNO_ ratio is calculated to be 1.78, suggesting that the hydrogenation of *NO proceeds through the NOH intermediate, rather than through the *HNO intermediate. Surprisingly, the ratio between the forward and backward rate constants (*k*_10_/*k*_11_: ~10^9^) is very high compared to their coverage ratio (1.78). The large difference is due to the high dissociation kinetics of *NOH (*k*_−10_) compared with *HNO (*k*_−11_). This is very much consistent with the previous trend observed from the relative energetics and activation barriers study, where we find hydrogenation at the O-centre is preferred over the N-centre.

However, hydrogenation at the N- and O- (of NO) is very important as hydrogenation at *N and O* sites will favour the formation of *NH_3_ and *N_2_ respectively, which in turn decides the product selectivity. The dissociation of *NOH leads to the formation of *N, whereas *HNO leads to the formation of *NH. *NH can be further hydrogenated for the formation of *NH_3_, whereas *N can undergo either hydrogenation or nitrogenation toward the formation of *NH_3_ and *N_2_ respectively. Thus, it is very important to understand that these hydrogenated pathways improve the product (*N_2_ vs. *NH_3_) selectivity.

Here Θ_NOH_/Θ_HNO_ ratio implies the partial pressure ratio of NO and H_2_. Several ratios of partial pressures of NO/H_2_ are used to understand their roles toward the product selectivity. The surface coverage ratios of Θ_NOH_/Θ_HNO_ are calculated for different partial pressure ratios of p_NO_/p_H2_. The calculated Θ_NOH_/Θ_HNO_ are 5.53 × 10^−02^, 1.59 × 10^−01^, 3.13 × 10^−01^, 6.41 × 10^−01^, 1.77 × 10^00^, 4.98 × 10^00^, 1.22 × 10^01^, 3.51 × 10^01^ and 3.92 × 10^02^ for 500, 40, 12, 4, 1, 0.25, 0.08, 0.025, 0.002 atmospheres of partial pressure (p_NO_/p_H2_) respectively. Therefore, *NOH coverage increases with increasing partial pressure of hydrogen, which further improves the product selectivity (*N_2_ formation).

From the DFT calculated parameters (Gibbs free energy and activation barriers) and the rate constant of the reaction analysis, we have already proposed that *N_2_ is the predominant product over *NH_3_ in the presence and absence of hydrogen. We find the spontaneous formation of *N_2_ with lower activation barrier and the exergonic behaviour of *NH_2_ dissociation with low activation barrier are the underlying reasons for the *N_2_ formation with higher selectivity. However, we wonder whether increasing hydrogen partial pressures can turn the selectivity toward *NH_3_? Therefore, after cropping the crucial steps from the complete microkinetic model, we have shown the effect of external hydrogen partial pressure toward the product selectivity. The small and effective microkinetic model is developed considering the following steps: (i)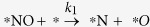, (ii) 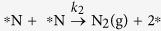, (iii) 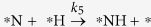, and (iv)
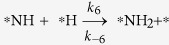
.

Therefore, the rate of formation of N_2_ and NH_2_ 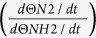 can be written as follows (see Supporting Information for details):


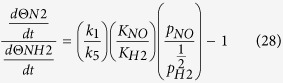


Several ratios of partial pressures of NO/H_2_ are used to understand their role toward the product selectivity. The calculated 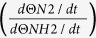 value is ≈6.77 × 10^35^ when the partial pressure ratio (p_H2_/p_NO_) of 500:1 is used. Therefore, our results suggest that even with extreme hydrogen partial pressures, N_2_ is the predominant product.

However, under the 1:1 partial pressure ratio of NO and H_2_, Θ_NOH_/Θ_HNO_ is found to be 1.78. Therefore, there could be a possibility of the reaction proceeding through the *HNO intermediate. Therefore, it is necessary to examine the extent of possibility for proceeding the reaction further from the *HNO intermediate. The ratio of rate constants (*k*_5_/*k*_2_) for 
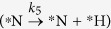
 and 
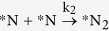
 is 1.58 × 10^09^, implying that the *NH formation is favourable over *N_2_. However, the ratio of rate constants (*k*_−5_/*k*_6_) for *NH dissociation 
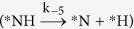
 and *NH_2_ formation 
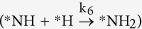
 is 4.17 × 10^06^, suggesting the tendency of *NH to decompose to *N instead of hydrogenation to *NH_2_. Therefore, it does not matter whether the reaction proceeds through the *HNO intermediate or not as it dissociates into *NH and *O (*HNO → *NH + *O). Interestingly, further hydrogenation on *NH is highly unfavourable (*NH + *H → *NH_2_) compared to *NH decomposition (*NH → *N + *H). Therefore, the Ni_85_ nanocluster shows excellent catalytic activity toward high product (N_2_) selectivity.

In the earlier sections, we have shown that *NOH coverage increases with increasing partial pressure of hydrogen. This in fact improves the formation of *N_2_. But with increasing of hydrogen partial pressures, does the formation of higher hydrogenated products (*HNOH and *H_2_NO) increase or not? Moreover, do such higher hydrogenated products also show greater selectivity towards the *N_2_ formation over *NH_3_ formation or not? The rate constants ratio [(*k*_14_ + *k*_15_)/*k*_16_] for the formation of *HNOH (*NOH + *H → *HNOH, *HNO + *H → *HNOH) and *H_2_NO is 5.88 × 10^06^, implying that *HNOH is the major product. Now, after N-O bond dissociation, *HNOH and *H_2_NO lead to the formation of *HN and *NH_2_, respectively. Interestingly, we already found *NH favours the formation of *N_2_ over *NH_3_.

Therefore, our study shows that the Ni_85_ nanocluster can efficiently and selectively reduce nitric oxide toward nitrogen even in the presence of high hydrogen partial pressure. Moreover, the most favourable pathway for *N_2_ formation is *NO → *N → *N_2_, whereas the least favourable pathway for *N_2_ formation is *NO → *ONNH → *N_2_O → *N_2_. Our results on the Ni-nanocluster are very much promising for product selectivity compared to previous experimental and theoretical reports on noble metal based catalysts (Pt, Pd, Rh, Ag, Pd).

## Discussions

DFT calculations have been performed to understand the nitric oxide reduction reaction over (111) facet of the octahedral nickel nanocluster (Ni_85_) enclosed by well-defined facets. Our results on the Ni-nanocluster show that direct N-O bond dissociation is thermodynamically as well as kinetically very much favourable even in the presence of hydrogen, which is completely opposite to earlier experimental and theoretical reports on noble metal based catalysts (Pt, Pd, Rh, Ag), where indirect N-O bond dissociation (from their hydrogenated products: NOH, HNO, HNOH, H_2_NO) is favourable over direct N-O bond dissociation. Hence, the product selectivity (N_2_ vs. N_2_O/NH_3_) increases on a Ni-nanocluster surface, whereas selectivity decreases on noble metal surfaces. The NO reduction reaction on Ni(111) bulk surfaces is reported by several groups, but no one has studied the reduction reaction in the presence of the hydrogen. Thus, a vis-à-vis comparison could not be made. The lower *N-O bond dissociation and N-N bond formation barriers make the Ni_85_ cluster an efficient and a selective catalyst toward NO reduction for N_2_ formation. In the presence of hydrogen, the reaction might proceed through *NOH or higher hydrogenated intermediates, but such intermediates easily dissociate into *NH or *N, which in turn favours *N_2_ formation over *NH_3_. Our microkinetic analysis shows that even with extreme hydrogen partial pressures, *N_2_ formation is favourable over *NH_3_. Thus, the product selectivity towards N_2_ does not change even under high hydrogen partial pressures on a Ni-nanocluster surface, which is again completely opposite to earlier experimental and theoretical reports on noble metal based catalysts (Pt, Pd, Rh, Ag). Hence, we report that nickel based nanoclusters could be very promising catalysts for the efficient and selective reduction of nitric oxide to nitrogen.

## Methods

The first-principles calculations are performed using the projected augmented wave (PAW) method^71^, as implemented in the Vienna ab initio simulation package (VASP)^72^,^73^,^74^. The exchange-correlation potential is described by using the generalized gradient approximation of Perdew-Burke-Ernzerhof (GGA-PBE)^75^. The projector augmented wave (PAW) method^71^ is employed to treat interactions between ion cores and valance electrons. The structure optimization is based on the conjugate gradient-minimization scheme under a spin polarization consideration. Our calculated magnetic value shows that each nickel has a magnetic moment of 0.71 *μ*_B_ which is in very much agreement with the previously reported value of 0.69 *μ*_B_^25^. For the consideration of van der Waals interactions, we have used Grimme’s D3-type of semiempirical method for dispersion energy correction^76^,^77^. But, we have listed the non-dispersion corrected values for a vis-à-vis comparison with earlier reports, as they have not included dispersion correction in their calculations. However, we have given both the dispersion-corrected and non-corrected values in the Supporting Information. A 22 × 22 × 22 Å^3^ cubic supercell is used to optimize the metal clusters to rule out the possibility of interaction of periodically repeated clusters. The Brillouin zone is sampled using gamma k-point (1 × 1 × 1). The total energy improved by 0.0001 eV when k-points are increased to 2 × 2 × 2. As the box size is quite high therefore we have used gamma point for our calculations. Plane wave with a kinetic energy cut off of 470 eV is used to expand the electronic wave functions. All the atoms are relaxed for the full structural relaxation. The climbing nudged elastic band (CI-NEB) method^78^ is used to locate the transition states. Six intermediate images are used in each CI-NEB pathway. Vibrational frequencies for the initial, transition and final states of the reactions are calculated and the transition state is confirmed from the presence of one imaginary frequency along the reaction coordinate. Zero-point energy (ZPE) is calculated from the following equation:


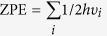


where *h* is the Planck constant and *υ*_*i*_ is the frequency of the *i*^*th*^ vibrational mode.

The reaction free energies (ΔG) and activation barriers (ΔG^‡^) are calculated using zero point energy (ZPE) and entropy corrections. The species are denoted with asterisk (*) while adsorbed on the surface.

## Additional Information

**How to cite this article**: Mahata, A. *et al*. Octahedral Ni-nanocluster (Ni_85_) for Efficient and Selective Reduction of Nitric Oxide (NO) to Nitrogen (N_2_). *Sci. Rep.* **6**, 25590; doi: 10.1038/srep25590 (2016).

## Supplementary Material

Supplementary Information

## Figures and Tables

**Table 1:** Rate constants (s^−1^) of the elementary reactions at different temperatures and here *k*_*i*_ and *k*_−*i*_ are for the forward and backward steps respectively.

Elementary reactions	300 K		350 K	400 K	450 K	500 K
	*k*_*i*_	*k*_−*i*_	*k*_*i*_	*k*_*i*_	*k*_*i*_	*k*_*i*_
*NO → *N + *O (*k*_1_)	4.77 × 10^−05^	3.88 × 10^−17^	1.57 × 10^−02^	1.23 × 10^00^	3.72 × 10^01^	5.76 × 10^02^
*N + *N → *N_2_ (*k*_2_)	5.07 × 10^−06^	1.24 × 10^−08^	1.89 × 10^−03^	1.63 × 10^−01^	5.28 × 10^00^	8.65 × 10^01^
*NO + *N → *N_2_O (*k*_3_)	1.39 × 10^−30^	3.68 × 10^−03^	1.98 × 10^−24^	8.27 × 10^−20^	3.30 × 10^−16^	2.54 × 10^−13^
*N_2_O → *N_2_ + *O (*k*_4_)	2.39 × 10^06^	9.11 × 10^−38^	2.64 × 10^07^	1.63 × 10^08^	6.80 × 10^08^	2.16 × 10^09^
*N + *H → *NH (*k*_5_)	8.02 × 10^03^	3.02 × 10^02^	1.49 × 10^05^	1.36 × 10^06^	7.69 × 10^06^	3.11 × 10^07^
*NH + *H → *NH_2_ (*k*_6_)	7.42 × 10^−05^	2.46 × 10^04^	2.32 × 10^−02^	1.76 × 10^00^	5.16 × 10^01^	7.79 × 10^02^
*NH_2_ + *H → *NH_3_ (*k*_7_)	3.68 × 10^04^	3.53 × 10^04^	6.33 × 10^05^	5.44 × 10^06^	2.94 × 10^07^	1.15 × 10^08^
*NO + *NH → *ONNH (*k*_8_)	4.41 × 10^−11^	1.52 × 10^13^	1.14 × 10^−07^	4.20 × 10^−05^	4.22 × 10^−03^	1.71 × 10^n^
*ONNH → *N_2_O + *H (*k*_9_)	2.70 × 10^−08^	1.95 × 10^−07^	2.78 × 10^−05^	5.87 × 10^−03^	3.02 × 10^−01^	7.94 × 10^00^
*NO + *H → *NOH (*k*_10_)	1.99 × 10^−09^	2.08 × 10^07^	2.83 × 10^−06^	6.66 × 10^−04^	4.72 × 10^−02^	1.44 × 10^00^
*NO + *H → *HNO (*k*_11_)	1.51 × 10^−18^	2.35 × 10^00^	4.36 × 10^−14^	9.82 × 10^−11^	4.04 × 10^−08^	5.03 × 10^−06^
*NOH → *N + *OH (*k*_12_)	1.25 × 10^−02^	2.69 × 10^−24^	1.97 × 10^00^	8.91 × 10^01^	1.75 × 10^03^	1.92 × 10^04^
*HNO → *NH + *O (*k*_13_)	1.08 × 10^00^	4.31 × 10^−31^	7.59 × 10^01^	1.87 × 10^03^	2.30 × 10^04^	1.73 × 10^05^
*NOH + *H → *HNOH (*k*_14_)	9.18 × 10^−09^	1.16 × 10^04^	9.90 × 10^−06^	1.90 × 10^−03^	1.15 × 10^−01^	3.08 × 10^00^
*HNO + *H → *HNOH (*k*_15_)	6.59 × 10^03^	6.28 × 10^08^	1.41 × 10^05^	1.43 × 10^06^	8.75 × 10^06^	3.78 × 10^07^
*HNO + *H → *H_2_NO (*k*_16_)	2.68 × 10^−03^	8.19 × 10^02^	4.73 × 10^−01^	2.33 × 10^01^	4.91 × 10^02^	5.67 × 10^03^
*HNOH → *NH + *OH (*k*_17_)	3.82 × 10^10^	1.62 × 10^−20^	1.00 × 10^11^	2.11 × 10^11^	3.81 × 10^11^	6.18 × 10^11^
*H_2_NO → *NH_2_ + *O (*k*_18_)	1.79 × 10^09^	8.28 × 10^−21^	6.86 × 10^09^	1.91 × 10^10^	4.31 × 10^10^	8.34 × 10^10^
*HNOH + *H → *H_2_NOH (*k*_19_)	7.11 × 10^−03^	6.57 × 10^−02^	1.12 × 10^00^	5.05 × 10^01^	9.91 × 10^02^	1.09 × 10^04^
*H_2_NO + *H → *H_2_NOH (*k*_20_)	4.66 × 10^08^	1.22 × 10^10^	2.08 × 10^09^	6.49 × 10^09^	1.59 × 10^10^	3.31 × 10^10^
*H_2_NOH → *NH_2_ + *OH (*k*_21_)	3.29 × 10^07^	3.53 × 10^−29^	2.19 × 10^08^	9.27 × 10^08^	2.89 × 10^09^	7.24 × 10^09^
*NO + *NOH → *ONNOH (*k*_22_)	2.18 × 10^−21^	1.54 × 10^n^	1.55 × 10^−16^	6.90 × 10^−13^	8.06 × 10^−03^	9.13 × 10^−08^
*ONNOH → *N_2_O + *OH (*k*_23_)	8.59 × 10^02^	1.13 × 10^−22^	2.61 × 10^04^	3.43 × 10^05^	2.58 × 10^06^	1.31 × 10^07^
*NO + *HNO → *ONNHO (*k*_24_)	8.48 × 10^−28^	5.70 × 10^02^	4.86 × 10^−22^	1.03 × 10^−17^	2.42 × 10^−14^	1.22 × 10^−11^
*ONNHO → *N_2_O + *OH (*k*_25_)	8.06 × 10^−03^	1.00 × 10^−30^	1.28 × 10^00^	5.85 × 10^01^	1.16 × 10^03^	1.28 × 10^04^
