# Supplementary material for: Octahedral Ni-nanocluster (Ni85) for Efficient and Selective Reduction of Nitric Oxide (NO) to Nitrogen (N2)
Source: Sci Rep. 2016 May 9;6:25590. doi: 10.1038/srep25590 (PMC4860637; doi:10.1038/srep25590)
Supplement: Supplementary Information [file srep25590-s1.pdf]

Octahedral Ni-nanocluster (Ni_{85}) for Efficient and Selective Reduction of Nitric Oxide (NO) to Nitrogen (N_2)

Arup Mahata,[†] Kuber Singh Rawat,[†] Indrani Choudhuri,[†] Biswarup Pathak, [†,#,*]

[†]Discipline of Chemistry, School of Basic Sciences, Indian Institute of Technology (IIT) Indore, Indore, M.P., India

[#]Center for Material Science and Engineering, Indian Institute of Technology (IIT) Indore, Indore, M. P., India

Email: biswarup@iiti.ac.in

Contents

S1: Microkinetic Analysis

S2: Microkinetic model considering the crucial steps

S3: Adsorption energy of the adsorbates with and without dispersion correction

S1: Microkinetic Analysis

A microkinetic analysis is developed from the DFT calculated values to give more insight into the coverage, reaction rate and the selectivity of different products. Elementary steps considered in the microkinetic model are summarized as following.

$NO (g) + * \rightarrow NO*$

$½ H_2 (g) + * \rightarrow H*$

$NO* + * \xrightarrow{k_1} N* + O*$

$N* + N* \xrightarrow{k_2} N_2 (g) + 2*$

$NO* + N* \underset{k_{-3}}{\overset{k_3}{\leftrightarrow}} N_2O* + *$

$N_2O* \xrightarrow{k_4} N_2 (g) + O*$

$N* + H* \xrightarrow{k_5} NH* + *$

$NH* + H* \underset{k_{-6}}{\overset{k_6}{\leftrightarrow}} NH_2* + *$

$NH_2* + H* \xrightarrow{k_7} NH_3 (g) + 2*$

$NO* + NH* \underset{k_{-8}}{\overset{k_8}{\leftrightarrow}} ONNH* + *$

$ONNH* + * \xrightarrow{k_9} N_2O* + H*$

$NO* + H* \underset{k_{-10}}{\overset{k_{10}}{\leftrightarrow}} NOH* + *$

$NO* + H* \underset{k_{-11}}{\overset{k_{11}}{\leftrightarrow}} HNO* + *$

$NOH* + * \xrightarrow{k_{12}} N* + OH*$

$HNO* + * \xrightarrow{k_{13}} NH* + O*$

$NOH* + H* \underset{k_{-14}}{\overset{k_{14}}{\leftrightarrow}} HNOH* + *$

$$HNO^* + H^* \underset{k_{-15}}{\overset{k_{15}}{\leftrightarrow}} HNOH^* + *$$

$$HNO^* + H^* \underset{k_{-16}}{\overset{k_{16}}{\leftrightarrow}} H_2NO^* + *$$

$$HNOH^* + * \xrightarrow{k_{17}} NH^* + OH^*$$

$$H_2NO^* + * \xrightarrow{k_{18}} NH_2^* + O^*$$

$$HNOH^* + H^* \xrightarrow{k_{19}} H_2NOH^* + *$$

$$H_2NO^* + H^* \xrightarrow{k_{20}} H_2NOH^* + *$$

$$H_2NOH^* + * \xrightarrow{k_{21}} NH_2^* + OH^*$$

$$NOH^* + NO^* \underset{k_{-22}}{\overset{k_{22}}{\leftrightarrow}} ONNOH^* + *$$

$$ONNOH^* + * \xrightarrow{k_{23}} N_2O^* + OH^*$$

$$HNO^* + NO^* \underset{k_{-24}}{\overset{k_{24}}{\leftrightarrow}} ONNHO^* + *$$

$$ONNHO^* + * \xrightarrow{k_{25}} N_2O^* + OH^*$$

The adsorption of NO and H_2 are highly exothermic and the process are assumed to be in equilibrium and are expected to be strongly adsorbed. The equilibrium constants are calculated using the following equation $K_{eq} = \exp[-(\Delta E_{ads}-T\Delta S)/k_BT]$, where ΔE_{ads} is the adsorption energy of NO and H_2, and ΔS is the entropy change of NO and H_2 upon adsorption, k_B is the Boltzmann's Constant and T is the temperature in Kelvin. The gas phase entropy is obtained from NIST database.[1] The other steps are described by forward and backward reaction rate. Rate constants are calculated using the following equation.

$$k_{i/-i} = \left(\frac{k_BT}{h}\right)\left(\frac{q_F}{q_I}\right)e^{-\Delta G^{\ddagger}/k_BT}$$

Where k_B is the Boltzmann constant, T is the temperature, h is the Plank constant. Here q_I and q_F are the vibrational partition functions for the initial and final state structures and $\Delta G^{\ddagger}$ is the Gibbs free energy barrier for the initial and final state of the elementary reaction. The vibrational partition functions were calculated using the following equation:

$$q = \sum_i \frac{1}{1 - e^{-h v_i / k_B T}}$$

where υ_i are the vibrational frequencies.

The backward reactions are not considered for all the exothermic forward reactions assuming the exothermic reactions are irreversible. The site balance of intermediate species included in the reaction is written in terms of coverage.

$\Theta_{NO} + \Theta_{NOH} + \Theta_{HNO} + \Theta_{HNOH} + \Theta_{HNNO} + \Theta_{H2NO} + \Theta_{H2NOH} + \Theta_{ONNOH} + \Theta_{ONNHO} + \Theta_{NH} + \Theta_{NH2} + \Theta_{N2O} + \Theta_{OH} + \Theta_{N} + \Theta_{O} + \Theta_{H} + \Theta_{N2} + \Theta_{NH3} + \Theta_{*} = 1$

The coverage of NO and H_2 are obtained by the following equations.

$\Theta_{NO} = p_{NO} K_{NO} \Theta_{*}$

$\Theta_{H} = p_{H2}^{1/2} K_{H2} \Theta_{*}$

The coverage of the other species is obtained by considering the steady-state approximation, where the rate of formation and rate of consumption of each species are assumed to be equal:

$d\Theta_{N2O}/dt = k_3\Theta_{NO}\,\Theta_{N} - k_4\Theta_{N2O} + k_9\Theta_{ONNH}\,\Theta_{*} + k_{23}\Theta_{ONNOH}\,\Theta_{*} + k_{25}\Theta_{ONNHO}\,\Theta_{*} = 0$

$d\Theta_{NH}/dt = k_{13}\Theta_{HNO}\,\Theta_{*} + k_{17}\Theta_{HNOH}\,\Theta_{*} - k_8\Theta_{NO}\,\Theta_{NH} + k_5\Theta_{N}\,\Theta_{H} - k_6\Theta_{NH}\,\Theta_{H} + k_{-8}\,\Theta_{ONNH} + k_{-6}\Theta_{NH2}\,\Theta_{*} = 0$

$d\Theta_{NH2}/dt = k_{18}\Theta_{H2NO}\,\Theta_{*} + k_{21}\Theta_{H2NOH}\,\Theta_{*} + k_6\Theta_{NH}\,\Theta_{H} - k_7\Theta_{NH2}\,\Theta_{H} - k_{-6}\Theta_{NH2}\,\Theta_{*} = 0$

$d\Theta_{NOH}/dt = k_{10}\Theta_{NO}\,\Theta_{H} - k_{12}\Theta_{NOH}\,\Theta_{*} - k_{14}\Theta_{NOH}\,\Theta_{H} + k_{-14}\Theta_{HNOH}\,\Theta_{*} - k_{22}\Theta_{NOH}\,\Theta_{NO} + k_{-22}\,\Theta_{ONNOH}\,\Theta_{*} - k_{-10}\Theta_{NOH}\,\Theta_{*} = 0$

$d\Theta_{HNO}/dt = k_{11}\Theta_{NO}\,\Theta_{H} - k_{13}\Theta_{HNO}\,\Theta_{*} - k_{16}\Theta_{HNO}\,\Theta_{H} + k_{-16}\Theta_{H2NO}\,\Theta_{*} - k_{24}\Theta_{HNO}\,\Theta_{NO} + k_{-24}\,\Theta_{ONNHO}\,\Theta_{*} - k_{-11}\Theta_{HNO}\,\Theta_{*} - k_{15}\Theta_{HNO}\,\Theta_{H} + k_{-15}\Theta_{HNOH}\,\Theta_{*} = 0$

$d\Theta_{HNOH}/dt = k_{14}\Theta_{NOH}\,\Theta_{H} - k_{17}\Theta_{HNOH}\,\Theta_{*} - k_{19}\Theta_{HNOH}\,\Theta_{H} + k_{15}\,\Theta_{HNO}\,\Theta_{H} - k_{-15}\Theta_{HNOH}\,\Theta_{*} - k_{-14}\Theta_{HNOH}\,\Theta_{*} = 0$

$d\Theta_{H2NO}/dt = k_{16}\Theta_{HNO}\,\Theta_{H} - k_{18}\Theta_{H2NO}\,\Theta_{*} - k_{20}\Theta_{H2NO}\,\Theta_{H} - k_{16}\Theta_{H2NO}\,\Theta_{*} = 0$

$d\Theta_{ONNH}/dt = k_{8}\Theta_{NO}\,\Theta_{NH} - k_{9}\Theta_{ONNH}\,\Theta_{*} - k_{-8}\,\Theta_{ONNH}\,\Theta_{*} = 0$

$d\Theta_{H2NOH}/dt = k_{19}\Theta_{HNOH}\,\Theta_{H} + k_{20}\Theta_{H2NO}\,\Theta_{H} - k_{21}\Theta_{H2NOH}\,\Theta_{*} = 0$

$d\Theta_{ONNOH}/dt = k_{22}\Theta_{NOH}\,\Theta_{NO} - k_{23}\Theta_{ONNOH}\,\Theta_{*} - k_{-22}\,\Theta_{ONNOH}\,\Theta_{*} = 0$

$d\Theta_{ONNHO}/dt = k_{24}\Theta_{HNO}\,\Theta_{NO} - k_{25}\Theta_{ONNHO}\,\Theta_{*} - k_{-24}\,\Theta_{ONNHO}\,\Theta_{*} = 0$

Now, solving the steady state equations of $d\Theta_{NOH}/dt$ and $d\Theta_{HNO}/dt$, the coverage ratio of Θ_{NOH} and Θ_{HNO} can be obtained as

$$\Theta_{NOH} = \frac{k_{-14}\Theta_{HNOH} + k_{10}K_{NO}K_{H2}p_{NO}p_{H2}^{1/2}}{k_{-10} + k_{12} + (k_{14}K_{H2}p_{H2}^{1/2}) + (k_{22}K_{NO}p_{NO}) - (\frac{k_{-22}k_{22}K_{NO}p_{NO}}{k_{23} + k_{-22}})}$$

$$\Theta_{HNO} = \frac{k_{-15}\Theta_{HNOH} + k_{11}K_{NO}K_{H2}p_{NO}p_{H2}^{1/2}}{k_{-11} + k_{13} + (k_{15}K_{H2}p_{H2}^{1/2}) + (k_{16}K_{H2}p_{H2}^{1/2}) + (k_{24}K_{NO}p_{NO}) + \left(\frac{k_{-16}k_{16}K_{H2}p_{H2}^{1/2}}{k_{-16} + k_{18} + k_{20}}\right) + (\frac{k_{-24}k_{24}K_{NO}p_{NO}}{k_{25} + k_{-24}})}$$

Our microkinetic analysis shows that adsorbed NO and adsorbed H are the most abundant species in the reaction medium. As k_{-8} and k_{-25} are very low compared to ($K_{NO} \times K_{H2}$), and Θ_{HNOH} is very low compared to Θ_{NO}, therefore

$$(k_{-14}\Theta_{HNOH} + k_{10}K_{NO}K_{H2}p_{NO}p_{H2}^{1/2}) \approx k_{10}K_{NO}K_{H2}p_{NO}p_{H2}^{1/2} \quad \text{and}$$

$$(k_{-15}\Theta_{HNOH} + k_{11}K_{NO}K_{H2}p_{NO}p_{H2}^{1/2}) \approx k_{11}K_{NO}K_{H2}p_{NO}p_{H2}^{1/2}$$

Thus, the final expressions of Θ_{NOH} and Θ_{HNO} are obtained as

$$\Theta_{NOH} = \frac{k_{10}K_{NO}K_{H2}p_{NO}p_{H2}^{1/2}}{k_{-10} + k_{12} + (k_{14}K_{H2}p_{H2}^{1/2}) + (k_{22}K_{NO}p_{NO}) - (\frac{k_{-22}k_{22}K_{NO}p_{NO}}{k_{23} + k_{-22}})}$$

$$\Theta_{HNO} = \frac{k_{11}K_{NO}K_{H2}p_{NO}p_{H2}^{1/2}}{k_{-11} + k_{13} + (k_{15}K_{H2}p_{H2}^{1/2}) + (k_{16}K_{H2}p_{H2}^{1/2}) + (k_{24}K_{NO}p_{NO}) + \left(\frac{k_{-16}k_{16}K_{H2}p_{H2}^{1/2}}{k_{-16} + k_{18} + k_{20}}\right) + (\frac{k_{-24}k_{24}K_{NO}p_{NO}}{k_{25} + k_{-24}})}$$

S2: Microkinetic model considering the crucial steps

$NO^* + * \xrightarrow{k_1} N^* + O^*$

$N^* + N^* \xrightarrow{k_2} N_2\ (g) + 2^*$

$N^* + H^* \xrightarrow{k_5} NH^* + *$

$NH^* + H^* \underset{k_{-6}}{\overset{k_6}{\leftrightarrow}} NH_2^* + *$

Now, applying the steady state for the intermediates, we can get

$d\Theta_N/dt = k_1\Theta_{NO}\,\Theta_* - k_2\Theta_N\,\Theta_N - k_5\Theta_N\,\Theta_H$

$d\Theta_{NH}/dt = k_5\Theta_{NO}\,\Theta_* - k_6\Theta_{NH}\,\Theta_H + k_{-6}\Theta_{NH2}\,\Theta_*$

$d\Theta_{NH2}/dt = k_6\Theta_{NH}\,\Theta_H - k_{-6}\Theta_{NH2}\,\Theta_*$

$d\Theta_{N2}/dt = k_2\Theta_N\,\Theta_N$

Solving the above equations, we can get

$$\frac{d\Theta N2/dt}{d\Theta NH2/dt} = \frac{k_1\Theta_{NO}\Theta_* - k_5\Theta_N\Theta_H}{k_5\Theta_N\Theta_H}$$

As both the $^*N_2$ and *NH formation is exergonic in nature, hence, the coverage of Θ_N is very low compared to $\Theta_{H.}$ Therefore, we can neglect Θ_N while compared to $\Theta_{H.}$ Thus,

$$\frac{d\Theta N2/dt}{d\Theta NH2/dt} = \frac{k_1\Theta_{NO}}{k_5\Theta_H} - 1$$

$$\frac{d\Theta N2/dt}{d\Theta NH2/dt} = \left(\frac{k_1}{k_5}\right)\left(\frac{K_{NO}}{K_{H2}}\right)\left(\frac{p_{NO}}{p_{H2}^{1/2}}\right) - 1$$

S3: Adsorption energy of the adsorbates with and without dispersion correction

Adsorbate	Adsorption Energy (eV)	
	With dispersion	Without dispersion
*NO	-4.23	-3.08
*N_2O	-1.15	-
*NOH	-4.99	-4.27
*HNO	-5.37	-4.56
*N_2	-1.37	-0.23
*NH	-5.89	-5.05
*NH_2	-4.82	-3.02
*NH_3	-1.49	-0.51
H_2NOH	-1.51	-
*ONNH	-3.92	-2.93
*ONNOH	-5.01	-5.07
*ONNHO	-3.30	-2.49
*HNOH	-4.44	-3.35
*H_2NO	-4.01	-1.36
*N	-6.74	-6.74
*O	-6.25	-5.02
*H	-3.36	-2.85

References:

1. http://webbook.nist.gov/chemistry/
